# Testing species hypotheses for *Fridericia magna*, an enchytraeid worm (Annelida: Clitellata) with great mitochondrial variation

**DOI:** 10.1186/s12862-020-01678-5

**Published:** 2020-09-14

**Authors:** Svante Martinsson, Mårten Klinth, Christer Erséus

**Affiliations:** Systematics and Biodiversity, Department of Biological and Environmental Sciences, University of Gothenburg, Box 463, SE-405 30 Göteborg, Sweden

**Keywords:** BPP, DNA-barcoding, Enchytraeidae, Haplowebs, Multispecies coalescence, Species delimitation

## Abstract

**Background:**

Deep mitochondrial divergences were observed in Scandinavian populations of the terrestrial to semi-aquatic annelid *Fridericia magna* (Clitellata: Enchytraeidae). This raised the need for testing whether the taxon is a single species or a complex of cryptic species.

**Results:**

A total of 62 specimens from 38 localities were included in the study, 44 of which were used for species delimitation. First, the 44 specimens were divided into clusters using ABGD (Automatic Barcode Gap Discovery) on two datasets, consisting of sequences of the mitochondrial markers COI and 16S. For each dataset, the worms were divided into six not completely congruent clusters. When they were combined, a maximum of seven clusters, or species hypotheses, were obtained, and the seven clusters were used as input in downstream analyses. We tested these hypotheses by constructing haplowebs for two nuclear markers, H3 and ITS, and in both haplowebs the specimens appeared as a single species. Multi-locus species delimitation analyses performed with the Bayesian BPP program also mainly supported a single species. Furthermore, no apparent morphological differences were found between the clusters. Two of the clusters were partially separated from each other and the other clusters, but not strongly enough to consider them as separate species. All 62 specimens were used to visualise the Scandinavian distribution, of the species, and to compare with published COI data from other *Fridericia* species.

**Conclusion:**

We show that the morphospecies *Fridericia magna* is a single species, harbouring several distinct mitochondrial clusters. There is partial genetic separation between some of them, which may be interpreted as incipient speciation. The study shows the importance of rigorous species delimitation using several independent markers when deep mitochondrial divergences might give the false impression of cryptic speciation.

## Background

Molecular studies of organismal DNA have proven many traditionally accepted species rank taxa to be complexes of morphologically similar, so called cryptic, species (see [1]). Examples are found in most animal groups e.g., [2], including segmented worms (Annelida) e.g., [3–5]. Mitochondrial markers, in particular, sometimes reveal distinct clusters of individuals within a genetically diverse but morphologically coherent assemblage of specimens, but testing such clusters as species hypotheses (putative cryptic species) in a standardised manner is not trivial. Methodological advances in species delimitation, e.g., approaches based on the multi-species coalescent (see [6, 7]) have been successfully incorporated in several studies on species delimitation in clitellate annelids e.g., [8–12]. A precise determination of species boundaries is important, not just for our understanding of the diversity of species, but also for their conservation e.g., [13–15]. There are also several cases where cryptic species within a species complex have been found to differ in important aspects of their biology, such as their response to pollutants [16, 17], predation risk [18], host preferences [19, 20], and habitat preferences [15, 21, 22].

**Fig. 1:**
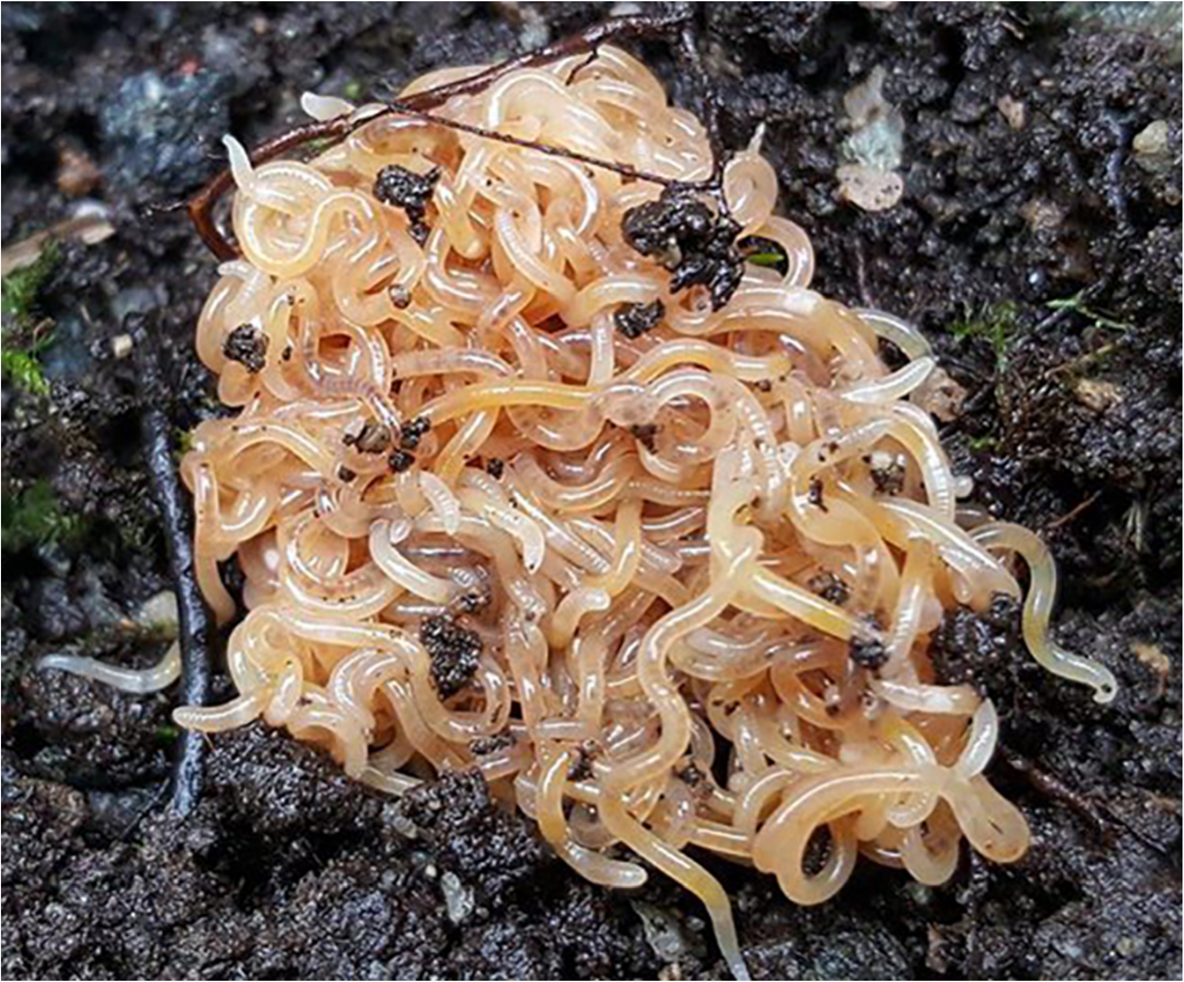
An aggregate of *Fridericia magna* Friend, 1899 (Clitellata: Enchytraeidae); from the collection site of specimens CE31735–36; Photo by Kate Michelsen

**Fig. 2:**
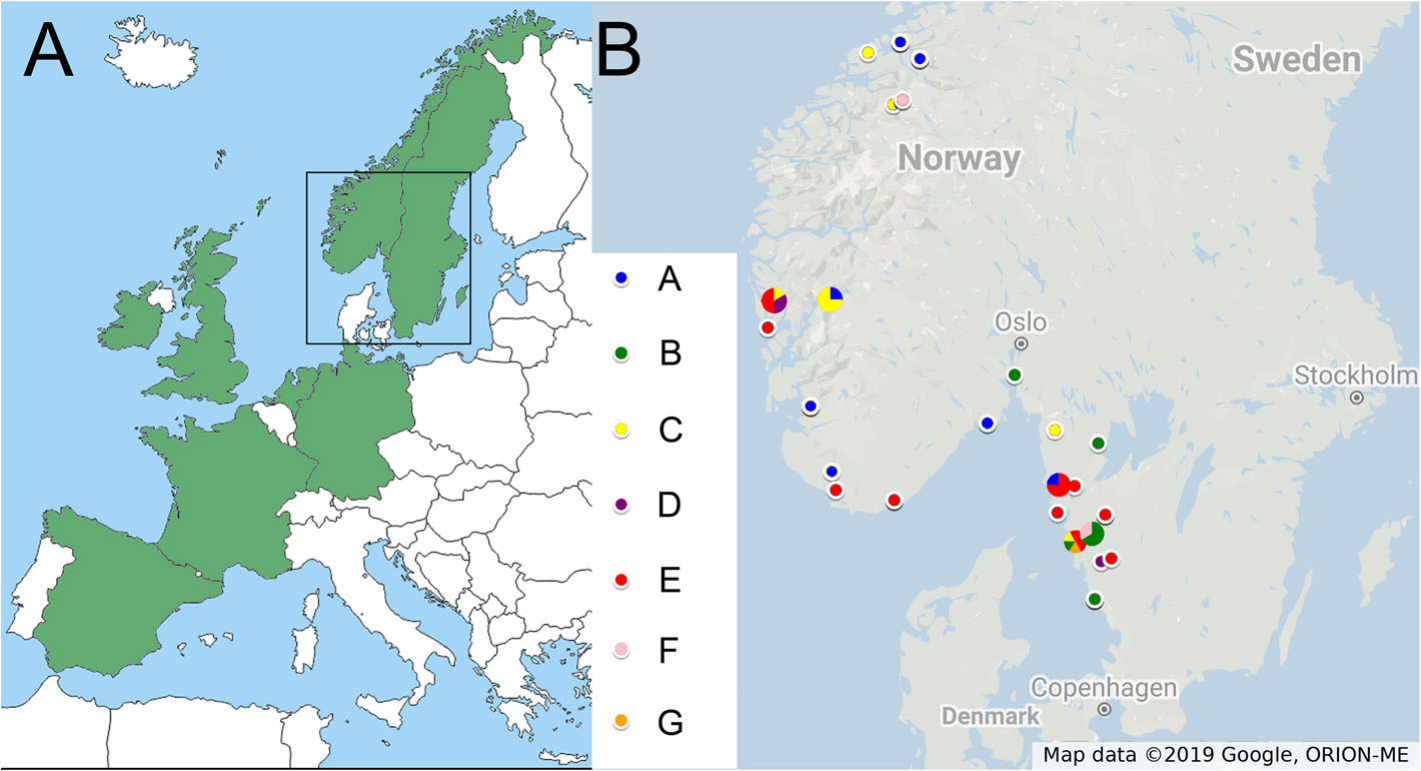
A Distribution of *Fridericia magna* in Europe, based on Schmelz [26], the subspecies *F. m.* ssp. *carpathica* Botea, 1973 from Bulgaria is excluded as it most likely represents a separate species, the rectangle indicate the position of zoomed in map in B. B. Distribution of *Fridericia magna* specimens used in this study, coloured according to mitochondrial lineages. For clarity, some closely situated localities have been combined. The map in A is based a map from D-maps (available at https://d-maps.com/carte.php?num_car=2232), the map in B is created in Google Maps, both maps were further edited in Adobe Photoshop. An interactive version of the map in B can be found at https://www.google.com/maps/d/edit?mid=1c4qeFc-BtsOtzf-QbuMS4P80pVo2cZ58&usp=sharing

During large-scale surveys of Clitellata in Scandinavia involving DNA barcoding (Erséus et al., ongoing work), we found deep divergence in the mitochondrial marker Cytochrome c Oxidase subunit I (COI) in the terrestrial worm, *Fridericia magna* Friend, 1899 [23] (family Enchytraeidae), suggesting that this taxon is a species complex. *Fridericia magna* (Fig. 1) is one of the largest species in the species-rich genus *Fridericia* Michaelsen, 1889 [24], and indeed one of the larger enchytraeids (see [25]). It is up to 50 mm long, and can consist of more than 90 segments [26], and is easily distinguished from congenerics by the combination of its large size, reduced chaetal numbers and red blood [26]. Originally described from the Lake district in England [23], it has a West-European distribution, with many twentieth century records from Spain in the south to Scotland in the north (Fig. 2A) [26]; a form from Romania, described as a subspecies of *F. magna* by Botea [27] is probably a different species [26]. A first specimen from Sweden was incorporated in a phylogenetic study by Erséus et al. ([28] as supplementary material). The species is mainly found in moist mineral soils, rich in organic material, and near rivers and lakes [26].

The aim of this study was to test whether the morphospecies *Fridericia magna* is a complex of several species or not, under the unified species concept [29], which postulates that the more lines of evidence for the existence of a “separately evolving metapopulation lineage”, the higher degree of corroboration in species delimitation. We sorted the specimens into potential species, based on genetic distances in the two mitochondrial markers COI and 16S rDNA, and tested these species hypotheses using two species criteria, the fields for recombination [30] using haplowebs [31], and the multispecies coalescent species concept [32] using BPP (Bayesian Phylogenetics and Phylogeography) [33, 34] on two nuclear markers Histone H3 (H3) and the Internal Transcribed Spacer region (ITS).

## Results

### Geographical distribution and habitats of the sampled material

A vast majority of our 38 Scandinavian sampling sites of *F. magna* are located in a coastal zone, extending to about 30 km from the sea, in south-western Sweden and then west- and northwards along the Norwegian coast to 63°N in Möre og Romsdal (Fig. 2B; Table S1). A single record was more inland, in the Swedish province of Dalsland near the large Lake Vänern. The habitats are of varying kinds, often soil with high organic contents, but in about half of the cases, the substrates were wet or fully submersed in water. All collection sites are located in regions of Sweden and Norway with high annual precipitation (> 900 mm per year) [35, 36].

### Specimens, DNA extraction and assembly

For all 62 specimens COI was obtained, 16S and H3 were successfully sequenced for 44 specimens, ITS was successfully sequenced for 42 specimens. The two COI alignments consist of 44 and 62 sequences respectively, the 16S alignment consists of 44 sequences, the COI alignments are 658 bp long, and the 16S alignment 483 bp. The ITS alignment is 950 bp long with 74 sequences, the H3 alignment 328 bp with 58 sequences; the higher numbers are due to the phasing of heterozygous sequences.

### Mitochondrial clustering and distance analysis

**Fig. 3:**
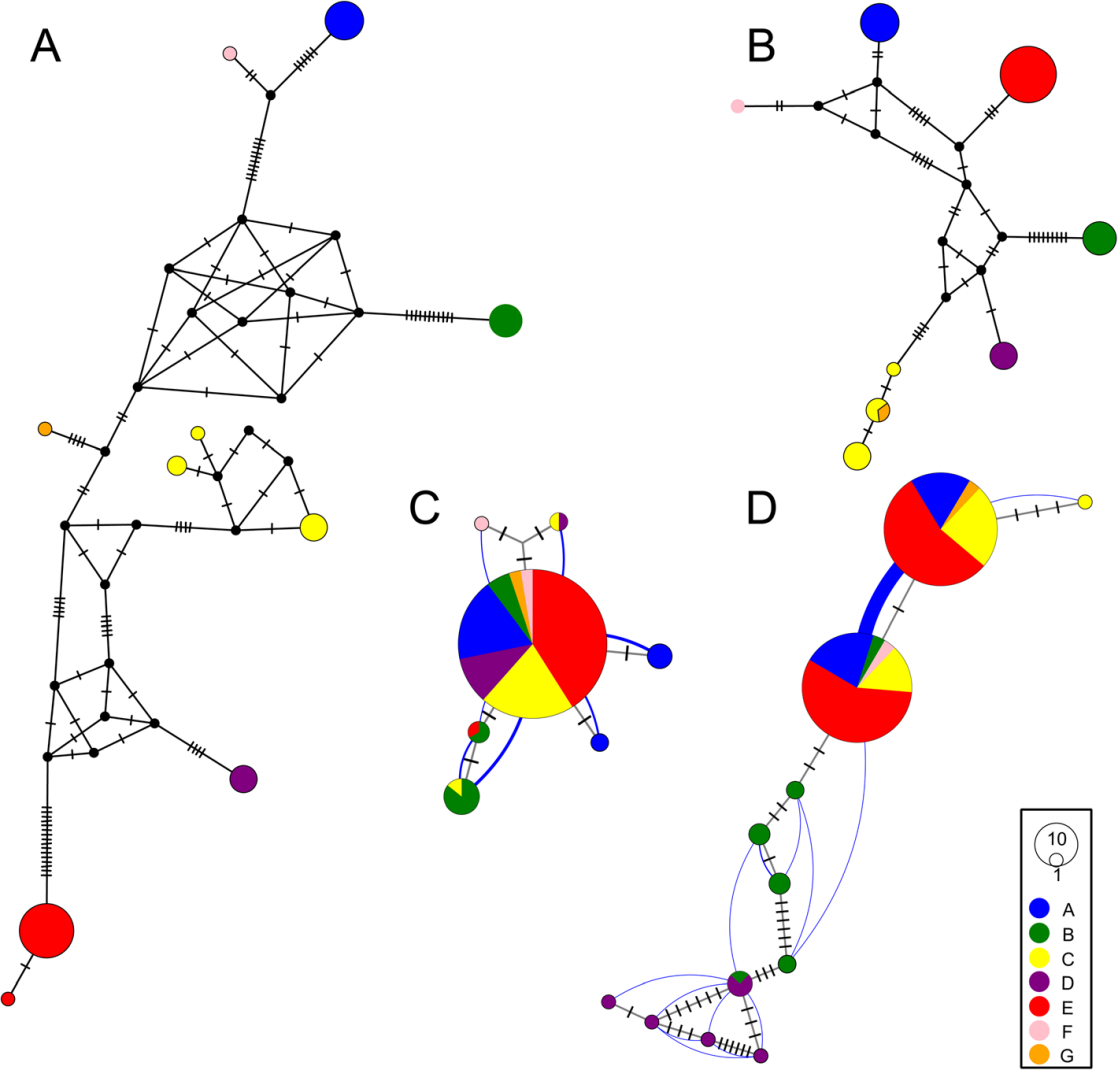
Medium joining haplotype networks for A: COI B: 16S C: H3 and D: ITS. The size of the circles is relative to the number of individuals sharing that haplotype, the colours correspond to the mitochondrial clusters, A-G, used as input in the species delimitation analysis, and the hatch marks correspond to the number of substitutions between haplotypes. The H3 and ITS networks are haplowebs, with the blue lines connecting haplotypes found in the same individual, and the line thickness correlating with the number of individuals having both haplotypes

**Fig. 4:**
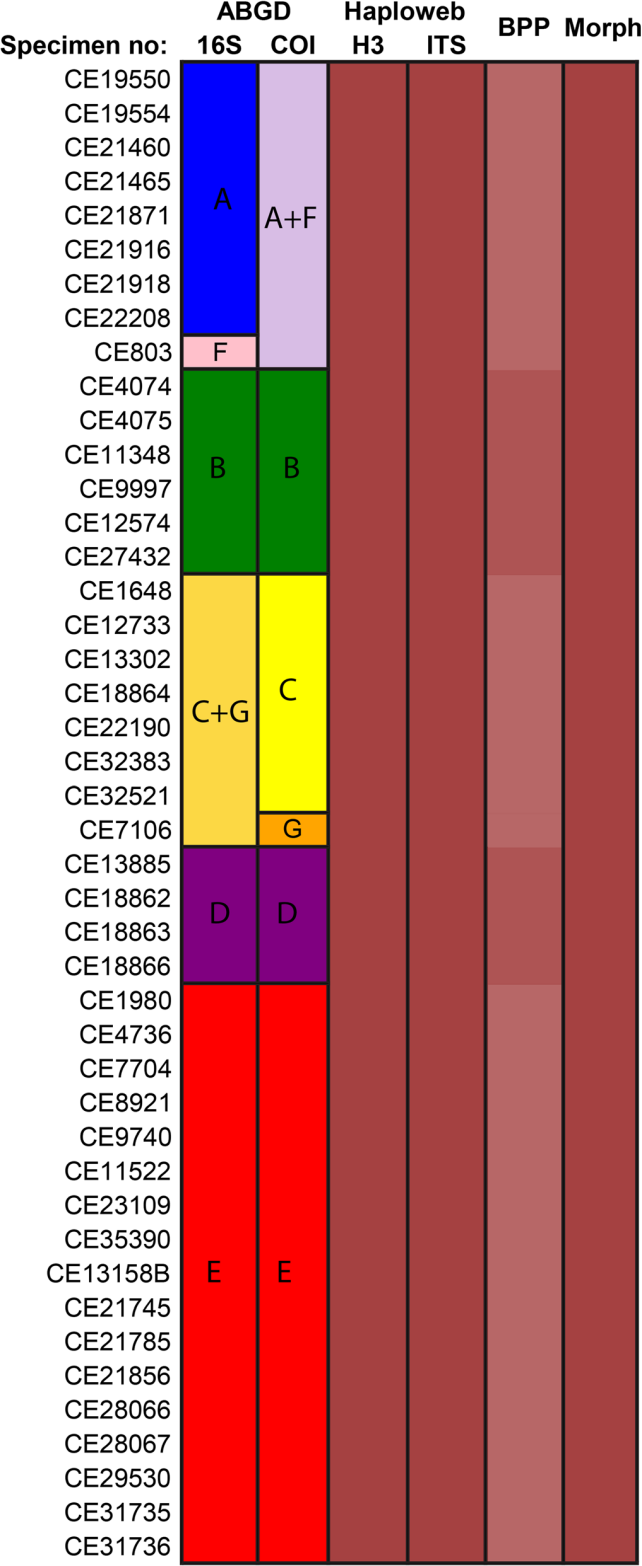
Summary of the results from the species delimitation. The coloured boxes show the delimited species of each analysis, including the morphological examination. For the BPP analyses the combined result of seven clusters (A-G) of the two ABGD analyses was used as the input species, the varying shades in the BPP column indicate the higher support for cluster B and D in one of the analyses

Uncorrected pairwise distances (p-distances) in the COI dataset vary between 0.0 and 8.8% (Fig. S1), and in the 16S dataset between 0.0 and 3.6%. The ABGD analyses divided both datasets into six clusters, but the clustering is not exactly the same in the two markers: one cluster found by the COI data is divided into two clusters by 16S, and vice versa, giving a maximum of seven possible clusters (named A-G; see Figs. 3 and 4), which were further tested in subsequent analyses. The maximum intra-cluster p-distances in COI vary between 0.0 and 1.1% (Table S2), and in 16S between 0.0 and 0.4% (Table S3), and the minimum inter-cluster p-distances in COI vary between 1.5 and 8.8% (Table S2), and in 16S between 0.0 and 3.4% (Table S3); the variation is visualised in the haplotype networks (Fig. 3A-B). At four collecting sites, two clusters were represented in sympatry (A + E, B + E, C + G, C + D, respectively; Table S1), and up to four clusters are found close to each other (at adjacent sites in Gothenburg; see Fig. 2B).

In the dataset with GenBank sequences of other *Fridericia* spp., the distances vary from 0.0 to 23.0% with a gap between 7.3 and 11.3% (Fig. S1). As the identification of many sequences on GenBank are doubtful, i.e., some sequences identified to the same species are widely separated, while some other sequences identified to different species are close together, we use this gap as an approximation of the separation of intra- and interspecies distances. The distances between many of the clusters of *F. magna* in COI are higher than the maximum intra-species distances in the GenBank dataset. However, in the COI gene tree with our *F. magna* specimens combined with data from GenBank (Fig. S2), most species (except *F. magna*) are represented by a single specimen or a few very similar sequences. This bias is likely to underestimate the intra-specific variation of the species represented in the GenBank data.

### Morphology

In total 31 specimens were studied, 25 of which were sexually mature. All six studied clusters (no specimen of cluster F was available) agreed with the description of *F. magna* in Schmelz, Collado [37]; body size being unusually large for *Fridericia*, and each spermatheca with two diverticula on the ampulla and two glands on the ectal duct, close to the ectal pore. However, we observed some slight differences in the chaetal pattern compared to the description in Schmelz, Collado [37]: in our material, the lateral chaetae were as commonly 1 as 2 (not mostly 2) per bundle, and the ventral chaetae were occasionally 1, but usually 2–4 per bundle anterior to the clitellum (not 3, or occasionally 4, 1 or 0 per bundle). Although the chaetal pattern was variable throughout our sample of specimens, we could not find any consistent differences between the clusters.

### Haplowebs

The haplowebs of the H3 and ITS datasets (Fig. 3C-D) both found only one species, as all haplotypes together form a single field for recombination. However, in the ITS haploweb (Fig. 3D) there is a tendency for cluster B and D to be separate, with only limited sharing of haplotypes between them and between B and the other clusters.

### Multi-locus species delimitation

Two of the three BPP analyses (A and B) found *F. magna* to be a single species, as none of the clusters were supported as separate species (Table 1). However in analysis C, clusters B and D were well supported as separate with a mean PP of > 0.95, in this analysis we used a population size prior assuming smaller genetic differences between random individuals from the population, than in the other two analyses. Based on the distances observed in the dataset, this prior is likely too small and therefore influences the analysis to accept more of the input species; the support for all input species is higher in analysis C than in A and B.

**Table 1:** List of species delimitations from the BPP analyses (A-C) and their mean posterior probabilities (PP). PP > 0.90 are marked in bold

Species delimitations	BPP analyses		
	A	B	C
A	0.492	0.576	0.815
B	0.727	0.744	**0.972**
C	0.537	0.570	0.772
D	0.701	0.720	**0.955**
E	0.438	0.512	0.738
F	0.596	0.625	0.829
G	0.323	0.376	0.529
ABCDEFG	0.251	0.231	0.000
CG	0.096	0.079	0.127
EG	0.093	0.104	0.149
AEG	0.071	0.037	0.022
AE	0.071	0.037	0.039
AG	0.061	0.074	0.092
DF	0.031	0.037	0.037
CEG	0.021	0.032	0.017
CFG	0.019	0.009	0.017
CF	0.017	0.028	0.032
FG	0.017	0.016	0.019
BF	0.016	0.020	0.026
CE	0.016	0.012	0.012
AEFG	0.011	0.003	0.002
AF	0.010	0.008	0.010

## Discussion

The results are summarised in Fig. 4. As neither the haplowebs, nor the majority of BPP analyses, or morphology support splitting *Fridericia magna* into several species, we conclude that it is a single species.

Despite the distinct mitochondrial clusters found within *F. magna***,** the consensus of the species delimitation methods support that all analysed specimens belong to a single species. Nevertheless, there is no completely randomized mixing of the clusters between the populations sampled. For ITS, clusters B and D were partly separated from the others, and from each other. This separation also got support by the BPP analysis C. This could be incipient speciation, which with time would result in three separate species. It is also possible that we observed despeciation [38], where the three groups had earlier been separated as separately evolving populations, i.e., species sensu de Queiroz [29], but have later started to interbreed to such a degree that the boundaries between them are dissolving, and they no longer can be considered separate species. However, we cannot rule out the possibility that the pattern of incomplete mixing observed is simply due to the limited amount of specimens included in this study, and that the pattern would disappear when more specimens are included.

*Fridericia magna* is one of several species of Clitellata where deep mt-divergence has been reported e.g., [10, 39–42], and large mitochondrial genetic distances seems to be common within clitellate species. However, there are also cases of species being delimited with small genetic distances between them [43–45]. In species with low dispersal rates there are more subdivisions compared to related species with higher dispersal rates [46]. Unfortunately, dispersal rates are poorly known for enchytraeids, and to our knowledge there are no estimates of such under field conditions. However, an estimate based on laboratory experiments, for *Cognettia sphagnetorum* s.lat. is less than 1 m per year [47]. In lumbricid earthworms the rate of active dispersal has been estimated to between 1.5 and 14 m per year depending on species and habitat [48]. Based on these estimates, it seems reasonable to assume that the dispersal rate for *F. magna* is a few meters per year. Such a low rate may be one of the factors in the evolutionary history of *F. magna* for the mitochondrial divergence observed, but it does not explain the apparent lack of geographic structure in our sample. *Fridericia magna* seems to be rather easily washed into streams and transported downstream, which could increase the mixing of the mitochondrial lineages.

One common explanation for observed mitochondrial divergence in clitellates is that it evolved during the Pleistocene glaciations when different populations were separated in different refugia. However, this has not been formally tested. *Fridericia magna* has a W European distribution, and it seems reasonable to assume that it survived the Pleistocene glaciations in refugia in SW Europe. Considering the recent history of colonization of the current soil fauna in Scandinavia since the end of the Weichselian glaciation about 12,500 years ago [49], it is most likely that the great mitochondrial variation in our material was largely established in the more southern parts of Western Europe.

In our analysis of the pairwise genetic distances of the *Fridericia spp*. from GenBank there is a clear gap between 7.3 and 11.3%. However, there are problems with the taxonomy of many of the sequences; in some clusters several species names are mixed, and another problem with this analysis is that for most species there is only a single sequence, or a few similar sequences available. These factors contribute to exaggeration of the global barcoding gap, and until a more complete dataset is available, with both more species and more sequences per species, it is hard to draw strong conclusions about the genetic variation within and between species of *Fridericia*.

The use of a single mitochondrial barcode, such as COI, is problematic, especially if a threshold distance is used as the main delimitation criteria, which was often the case in early DNA-barcoding literature e.g., [50, 51], and which is still in practice in the Barcode Index Number (BIN) System used by the Barcoding of Life Data System (BOLD) [52]. Instead it now seems that each case is unique, and a proper species delimitation analysis, including more data, is needed to establish the species boundaries. We urge taxonomists to test all species hypotheses using an integrative approach, involving also nuclear data, as well as organismal-level evidence, such as morphology, physiology, behaviour, life history traits, if possible.

## Conclusions

We find no evidence that *Fridericia magna* specimens collected in SW Scandinavia, despite their great genetic variation, belong to a complex of cryptic species. The study underpins the problem with using only a single mitochondrial marker (a DNA barcode) together with a global threshold value in species delimitation (see [53]), instead each case should been seen as unique.

## Methods

### Specimens, DNA extraction and assembly

In total, 62 specimens of the morphospecies *Fridericia magna*, collected in Norway and Sweden (Fig. 2) between 2004 and 2016 (see Table 2, and Table S1 for details) are included in the study. For 19 of them only COI was sequenced, and these are not included in the species delimitation analyses. It can be noted that, at some of the sampling sites, numerous specimens had evidently been washed out from their natural habitats by heavy rain, and were found in aggregations (Fig. 1) in small temporary water bodies (puddles or flooding streams). One specimen (CE 23109) was found in stomach contents of a juvenile Atlantic Salmon (*Salmo salar*) caught in Bodeleån River, Uddevalla, Bohuslän, Sweden.

**Table 2:** Specimens, with GenBank accession numbers; accession numbers in bold face are newly generated; more details in Supplementary Table S1. Morphologically examined specimens are indicated by an asterix (*)

Specimen no.	Cluster	Country	GenBank Accession no.					
			COI	16S	ITS		H3	
CE18491	A	Norway	**MT609948**	**–**	**–**	**–**	**–**	**–**
CE18492	A	Norway	**MT609951**	**–**	**–**	**–**	**–**	**–**
CE18493	A	Norway	**MT609946**	**–**	**–**	**–**	**–**	**–**
CE19550*	A	Norway	**MT580300**	**MT602462**	**MT603764**	**–**	**MT601975**	**–**
CE19551	A	Norway	**MT609947**	**–**	**–**	**–**	**–**	**–**
CE19554*	A	Norway	**MT580301**	**MT602464**	**MT603765**	**–**	**MT601976**	**–**
CE21460	A	Norway	**MT580303**	**MT602467**	**MT603768**	**–**	**MT601978**	**MT601979**
CE21465	A	Norway	**MT580304**	**MT602466**	**MT603769**	**–**	**MT601980**	**MT601981**
CE21871*	A	Norway	**MT580308**	**MT602468**	**MT603774**	**MT603775**	**MT602002**	**MT602003**
CE21916*	A	Norway	**MT580309**	**MT602463**	**MT603776**	**–**	**MT601985**	**MT601986**
CE21918*	A	Norway	**MT580310**	**MT602469**	**MT603777**	**MT603778**	**MT602004**	**–**
CE21921	A	Norway	**MT580340**	**–**	**–**	**–**	**–**	**–**
CE22208*	A	Norway	**MT580312**	**MT602465**	**MT603781**	**MT603782**	**MT601987**	**MT601988**
CE4738	A	Sweden	**MT580332**	**–**	**–**	**–**	**–**	**–**
CE12574*	B	Norway	**MT580290**	**MT602473**	**MT603751**	**MT603752**	**MT601963**	**–**
CE11348*	B	Sweden	**MT580288**	**MT602470**	**MT603747**	**MT603748**	**MT601960**	**MT601961**
CE27432*	B	Sweden	**MT580314**	**MT602475**	**MT603785**	**MT603786**	**MT602008**	**MT602009**
CE4074*	B	Sweden	**MT580323**	**MT602471**	**MT603799**	**MT603800**	**MT601991**	**–**
CE4075*	B	Sweden	**MT580324**	**MT602474**	**MT603801**	**MT603802**	**MT601992**	**MT601993**
CE4076	B	Sweden	**MT580331**	**–**	**–**	**–**	**–**	**–**
CE9996	B	Sweden	**MT580334**	**–**	**–**	**–**	**–**	**–**
CE9997*	B	Sweden	**MT580329**	**MT602472**	**MT603813**	**MT603814**	**MT602000**	**MT602001**
CE12733*	C	Norway	**MT580291**	**MT602497**	**MT603739**	**MT603740**	**MT601964**	**–**
CE12734	C	Norway	**MT609954**	**–**	**–**	**–**	**–**	**–**
CE13302*	C	Norway	**MT580293**	**MT602498**	**MT603755**	**–**	**MT601966**	**–**
CE18864*	C	Norway	**MT580298**	**MT602504**	**–**	**–**	**MT601971**	**MT601972**
CE22190*	C	Norway	**MT580311**	**MT602499**	**MT603779**	**MT603780**	**MT602005**	**MT602006**
CE32383*	C	Norway	**MT580320**	**MT602500**	**MT603795**	**MT603796**	**MT602014**	**–**
CE32521*	C	Norway	**MT580321**	**MT602502**	**MT603745**	**MT603746**	**MT601990**	**–**
CE1648	C	Sweden	**MT580295**	**MT602501**	**MT603757**	**–**	**MT601968**	**–**
CE18862*	D	Norway	**MT580296**	**MT602477**	**MT603760**	**MT603759**	**MT601969**	**–**
CE18863*	D	Norway	**MT580297**	**MT602478**	**MT603762**	**MT603763**	**MT601970**	**–**
CE18865	D	Norway	**MT580335**	**–**	**–**	**–**	**–**	**–**
CE18866*	D	Norway	**MT580299**	**MT602479**	**–**	**–**	**MT601973**	**MT601974**
CE13885*	D	Sweden	**MT580294**	**MT602476**	**MT603756**	**–**	**MT601967**	**–**
CE13158B*	E	Norway	**MT580292**	**MT602493**	**MT603753**	**MT603754**	**MT601965**	**–**
CE18454	E	Norway	**MT609953**	**–**	**–**	**–**	**–**	**–**
CE21745	E	Norway	**MT580305**	**MT602481**	**MT603770**	**MT603771**	**MT601982**	**–**
CE21785	E	Norway	**MT580306**	**MT602487**	**MT603772**	**MT603773**	**MT601983**	**–**
CE21786	E	Norway	**MT580339**	**–**	**–**	**–**	**–**	**–**
CE21856	E	Norway	**MT580307**	**MT602482**	**MT603741**	**MT603742**	**MT601984**	**–**
CE28066	E	Norway	**MT580315**	**MT602490**	**MT603787**	**MT603788**	**MT602010**	**–**
CE28067	E	Norway	**MT580316**	**MT602488**	**MT603789**	**MT603790**	**MT602011**	**–**
CE29530	E	Norway	**MT580317**	**MT602494**	**MT603791**	**MT603792**	**MT602012**	**–**
CE31735*	E	Norway	**MT580318**	**MT602483**	**MT603743**	**MT603744**	**MT601989**	**–**
CE31736	E	Norway	**MT580319**	**MT602495**	**MT603793**	**MT603794**	**MT602013**	**–**
CE11522*	E	Sweden	**MT580289**	**MT602485**	**MT603749**	**MT603750**	**MT601962**	**–**
CE1980*	E	Sweden	**MT580302**	**MT602480**	**MT603766**	**MT603767**	**MT601977**	**–**
CE1981	E	Sweden	**MT580338**	**–**	**–**	**–**	**–**	**–**
CE23109*	E	Sweden	**MT580313**	**MT602489**	**MT603783**	**MT603784**	**MT602007**	**–**
CE35390	E	Sweden	**MT580322**	**MT602491**	**MT603797**	**MT603798**	**MT602015**	**MT602016**
CE4736*	E	Sweden	**MT580325**	**MT602486**	**MT603803**	**MT603804**	**MT601994**	**–**
CE4737	E	Sweden	**MT580336**	**–**	**–**	**–**	**–**	**–**
CE4739	E	Sweden	**MT580337**	**–**	**–**	**–**	**–**	**–**
CE7704*	E	Sweden	**MT580326**	**MT602484**	**MT603806**	**MT603807**	**MT601996**	**–**
CE7715	E	Sweden	**MT580333**	**–**	**–**	**–**	**–**	**–**
CE8921*	E	Sweden	**MT580327**	**MT602495**	**MT603809**	**MT603810**	**MT601998**	**–**
CE9740	E	Sweden	**MT580328**	**MT602492**	**MT603811**	**MT603812**	**MT601999**	**–**
CE18487	F	Norway	**MT609949**	**–**	**–**	**–**	**–**	**–**
CE18488	F	Norway	**MT609952**	**–**	**–**	**–**	**–**	**–**
CE803	F	Sweden	GU901804 ^**1**^	GU902066 ^1^	**MT603808**	–	MN248702 ^2^	**MT601997**
CE7106*	G	Sweden	**MT580330**	**MT602503**	**MT603805**	**–**	**MT601995**	–

^1^From Erséus et al. [28]; ^2^From Schmelz et al. [54]

DNA was extracted from the posterior ends of ethanol-preserved worms, while the anterior parts of the same worms were either mounted in Canada balsam, or stored in 80% ethanol, to serve as physical vouchers. DNA was extracted using either Qiagen DNeasy Blood & Tissue Kit or Epicentre QuickExtract DNA Extraction Solution 1.0, following the manufacturer’s instructions. Four markers, the mitochondrial Cytochrome c oxidase subunit I (COI) gene, the mitochondrial ribosomal 16S gene, the complete nuclear ribosomal Internal Transcribed Spacer (ITS) region, and the nuclear gene Histone H3 (H3), were amplified using primers and PCR programmes listed in Table S4. Sequencing was carried out by Macrogen Inc. (Seoul, Korea) or Eurofins MWG Operon (Ebersberg, Germany), 9 specimens were handled by the Canadian Centre for DNA Barcoding (CCDB) (Guelph, Canada), with data stored at the Barcode of Life Datasystems (BOLD), these are part of the 19 worms with COI data only (see above). As specified in Table 2 and Table S1, two sequences from Erséus et al. [28], and one from [54] were downloaded from GenBank. Moreover, for two specimens (CE18864, CE18866), attempts at sequencing ITS were unsuccessful. Sequences were assembled in Geneious Pro v. 7.1 (Biomatters Ltd.; http://www.geneious.com) and aligned separately for each gene using MAFFT v7.017 [55], as implemented in Geneious Pro v. 7.1, using the auto-algorithm and default settings. For COI two datasets were created, one with the 44 species for which all markers were attempted to be sequenced, and one with all 62 COI sequences. A separate dataset consisting of all 129 *Fridericia* COI sequences available on GenBank (accessed 2 Jun 2020) was also assembled.

In the H3 and ITS datasets, several individuals showed clear signs of heterozygosity, i.e., distinct double peaks at certain positions in the sequencing chromatograms. Due to this, we separated H3 and ITS alleles using the PHASE algorithm [56, 57] as implemented in DNAsp v. 5.10 [58]. The phasing was run for 200 iterations after 100 initial burn-in iterations, with a thinning interval of 1 using default settings. For homozygous specimens only one of the two identical alleles was kept. Furthermore, two individuals had length variation in ITS, for these specimens phase determination was performed by direct sequencing [59] with the help of Champuru v1.0 [60], available online at http://www.mnhn.fr/jfflot/champuru/. The phased datasets were used in all subsequent analyses.

All new sequences produced in this study are deposited in GenBank, and the vouchers are deposited in either the Swedish Museum of Natural History (SMNH), Stockholm, Sweden, or the University Museum of Bergen (ZMBN), Bergen, Norway (accession numbers in Table S1).

### Mitochondrial clustering and distance analysis

The *F. magna* specimens were clustered into groups using the two mitochondrial markers. Uncorrected genetic p-distances were calculated for the mitochondrial COI and 16S datasets in MEGA X [61], using pairwise deletion for missing data. The distances were then analysed with the online version of ABGD (Automatic Barcode Gap Discovery [62]; available at http://wwwabi.snv.jussieu.fr/public/abgd/abgdweb.html), with default settings, to divide the specimens into potential species. The latter were then subsequently tested using the nuclear markers (see below). The variation in COI and 16S was visualized by haplotype networks created in PopART v1 [63] using medium joining [64]; sites with missing data or gaps were masked and not included in the networks. Uncorrected genetic p-distances were also calculated for the dataset of COI sequences from GenBank, and these were compared with the distance of the *F. magna* COI dataset and summarised in a histogram. A gene tree of all COI sequences, both from GenBank and the sequences of *F. magna*, combined was estimated with ML using phyML 3.0 [65]; Smart Model Selection [66] with Bayesian Information criterion was used for automatic model selection; and Subtree Pruning and Regrafting were used for tree improvement. Branch support was calculated with the SH-like (Shimodaira-Hasegawa test-like) approximative likelihood ratio test (aLRT) [67]. The tree was rooted using midpoint rooting and drawn in FigTree 1.4.2 [68] and further edited in Adobe Illustrator.

### Morphology

Immature and sexually mature specimens from six of the seven potential species were examined morphologically, excluding cluster F for which we had no voucher (Tables 2, S1; both specifying which specimens that were mounted and examined). The characters examined were body size, chaetal formula and spermathecal morphology, other internal characters were difficult to observe in the whole-mounted material, due to the size of the worms. The morphology was compared to the description in Schmelz, Collado [37].

### Haplowebs

To find the fields for recombination, i.e., groups of specimens that share a set of haplotypes connected by heterozygous individuals [30], haplowebs [31] were constructed for the nuclear ITS and H3 datasets with HaplowebMaker [69], available online at https://eeg-ebe.github.io/HaplowebMaker/, constructing median joining networks [64], and treating indels as a 5th character state. The haplotypes were coloured according to the mitochondrial clusters. Haplowebs visualise the fields for recombination by connecting haplotypes that are found within the same individual.

### Multi-locus species delimitation

To test the potential species, under the multispecies coalescent species concept [32], multi-locus species delimitation was performed using BPP v.3.3 [34, 70], for the two nuclear markers H3 and ITS. As the COI and 16S datasets were used for the initial sorting of specimens into groups, and therefore match the groups found by design, they were not included in the analyses. Joint Bayesian species delimitations and species tree estimations [33, 70, 71] were conducted; thereafter, three analyses (A-C) with different population size (estimated by θ) and divergence time (τ0) priors were performed, using the same settings and priors as in Martinsson, Erséus [43] (A: θ 2, 400, τ0 2, 200; B: θ 2, 1000, τ0 2, 200;C: θ 2, 2000, τ0 2, 200); all are diffuse priors with α = 2, the difference between the analyses is in the population size prior θ, which reflect the genetic distance between two sequences sampled at random from the population [34]. In analysis A we used a large prior (2/400 = 0.005), in C a small prior, and with an intermediate prior in analysis B. The analyses were each run for 200,000 generations, discarding the first 4000 as burn-in, and all analyses were performed three times to confirm consistency between runs. We considered species delimited with a PP (posterior probability) > 0.90 in all analyses to be well supported.

## Supplementary information

**Additional file 1 Fig. S1.** Histogram of uncorrected pairwise genetic distances given in percent for COI sequences of Fridericia spp. sequences from GenBank and *F. magna* from this study.

**Additional file 2 Fig. S2.** COI gene tree of our Fridericia magna specimens and COI sequences of Fridericia spp. from GenBank. The tree is estimated with Maximum Likelihood in PhyML. Scale show expected number of changes per site.

**Additional file 3: Table S1.** Specimens included in the study, with individual specimen numbers, collection data, museum voucher numbers, and GenBank accession numbers, accession numbers in bold are newly generated in this study. *Morphologically examined specimens mounted on slides. **Table S2.** Uncorrected pairwise genetic distances (p-dist) in COI for the clusters of *Fridericia magna*, the intraclustal distances are given as the largest p-dist and the intercluster distances as the smallest p-dist. **Table S3**, Uncorrected pairwise genetic distances (p-dist) in 16S for the clusters of *Fridericia magna*, the intraclustal distances are given as the largest p-dist and the intercluster distances as the smallest p-dist. **Table S4.** Primers and programs used for amplification and sequencing of fragments of the mitochondrial 16S and COI and nuclear ITS and H3 markers.

## Data Availability

The specimens included in this work are deposited in the Swedish Museum of Natural History (SMNH), Stockholm, Sweden, and the University Museum of Bergen (ZMBN), Bergen, Norway; accession numbers in Table S1. The DNA sequence data generated for this article are available on GenBank; see Table 2 for accession numbers. The DNA sequence alignments used in the analyses, as well as files associated with the BPP analyses, have been deposited on GitHub (https://github.com/Svante-Martinsson/Fridericia_magna), and an interactive version of the distribution map in Fig. 2 B is available on https://www.google.com/maps/d/edit?mid=1c4qeFc-BtsOtzf-QbuMS4P80pVo2cZ58&usp=sharing

## Abbreviations

16S: 16S ribosomal DNA
ABGD: Automatic Barcode Gap Discovery
aLRT: approximative likelihood ratio test
BIN: Barcode Index Number
BOLD: Barcoding of Life Data System
BPP: Bayesian Phylogenetics and Phylogeography
CCDB: Canadian Centre for DNA Barcoding
COI: Cytochrome c Oxidase subunit I
DNA: Deoxyribonucleic acid
E: East
H3: Histone H3
ITS: Internal Transcribed Spacer
ML: Maximum Likelihood
N: North
no.: number
p-distances: pairwise distances
SH-like: Shimodaira-Hasegawa test-like
SMNH: Swedish Museum of Natural History
SW: South-westen
W: West
ZMBN: University Museum of Bergen
