# Supplementary material for: Testing species hypotheses for Fridericia magna, an enchytraeid worm (Annelida: Clitellata) with great mitochondrial variation
Source: BMC Evol Biol. 2020 Sep 14;20:116. doi: 10.1186/s12862-020-01678-5 (PMC7488859; doi:10.1186/s12862-020-01678-5)
Supplement: Supplementary file 1 — Additional file 1 Fig. S1. Histogram of uncorrected pairwise genetic distances given in percent for COI sequences of Fridericia spp. sequences from GenBank and F. magna from this study. [file 12862_2020_1678_MOESM1_ESM.pdf]

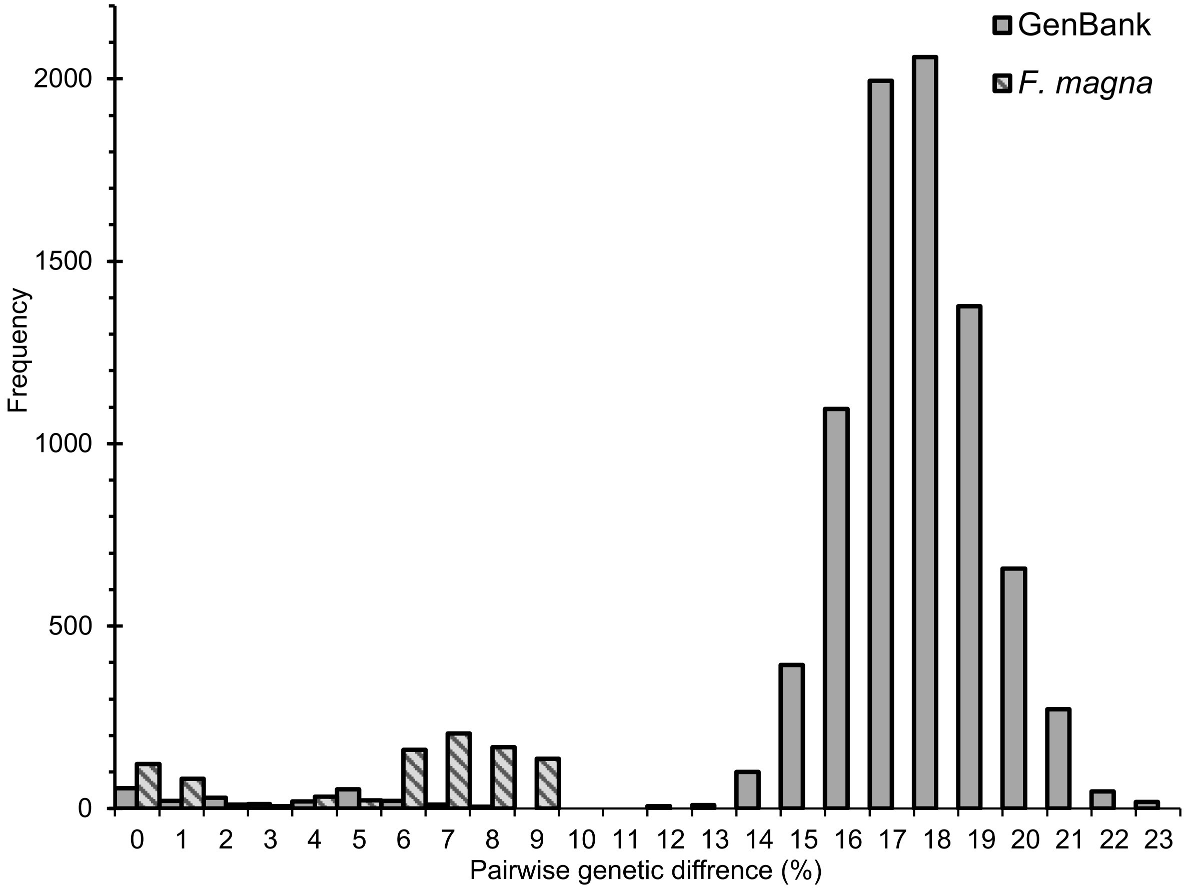

Supplementary Figure S1. Histogram of uncorrected pairwise genetic distances given in percent for COI sequences of *Fridericia spp*. sequences from GenBank and *F. magna* from this study.
