# Supplementary figures and images for: Testing species hypotheses for Fridericia magna, an enchytraeid worm (Annelida: Clitellata) with great mitochondrial variation
Source: BMC Evol Biol. 2020 Sep 14;20:116. doi: 10.1186/s12862-020-01678-5 (PMC7488859; doi:10.1186/s12862-020-01678-5)

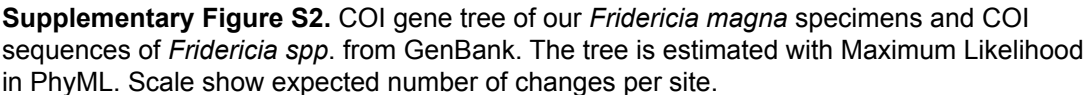

Supplement: Supplementary file 2 — Additional file 2 Fig. S2. COI gene tree of our Fridericia magna specimens and COI sequences of Fridericia spp. from GenBank. The tree is estimated with Maximum Likelihood in PhyML. Scale show expected number of changes per site. [file 12862_2020_1678_MOESM2_ESM.pdf]
